# Retraction: Open-Loop and Closed-Loop Neuromodulation Across Neurological Disorders Toward Personalized Brain Stimulation: A Narrative Review

**DOI:** 10.7759/cureus.r263

**Published:** 2026-11-05

**Authors:** Meet Popatbhai Kachhadia, Imad Sibhai, Rushi Vaghela, Usmaan Topiwala, Juber D Shaikh, Michelle Tsai, Nirali Borad, Michael Simon, Hasan Ilyas, Touqir Zahra

**Affiliations:** 1 Neurology, Florida Atlantic University Charles E. Schmidt College of Medicine, Boca Raton, USA; 2 Medical Education, Smt. N. H. L. Municipal Medical College, Ahmedabad, IND; 3 Internal Medicine, Smt. N. H. L. Municipal Medical College, Ahmedabad, IND; 4 General Medicine, Smt. N. H. L. Municipal Medical College, Ahmedabad, IND; 5 Neurology, Prisma Health/University of South Carolina, Columbia, USA; 6 Internal Medicine, Florida Atlantic University Charles E. Schmidt College of Medicine, Boca Raton, USA; 7 Pediatrics, WVU Thomas Memorial Hospital, Charleston, USA

This article has been retracted by the Editor-in-Chief. An editorial review found that the references cited do not support the statements to which they are attached and that the sources used to prepare the article could not be established. The authors did not respond to repeated requests by the journal.

